# Structure and Dynamics of the Deprotonated Demethoxycurcumin and Bisdemethoxycurcumin Anions

**DOI:** 10.1021/acs.jpca.5c08039

**Published:** 2026-02-12

**Authors:** Jemma A. Gibbard

**Affiliations:** Department of Chemistry, 3057 Durham University, Durham DH1 3LE, United Kingdom

## Abstract

The curcuminoids are polyphenols found in the spice turmeric, where its principal polyphenol, curcumin, has been attributed with many of turmeric’s beneficial therapeutic properties. However, as curcumin has a low bioavailability, other curcuminoids, which have fewer terminal methoxy substituents and have been reported to have a higher bioavailability, have been investigated as possible therapeutics. In this article, we report the results of the first gas-phase ion spectroscopy study of the deprotonated anionic forms of two prevalent curcuminoids: demethoxycurcumin and bisdemethoxycurcumin. The results indicate that the deprotonated curcuminoid anions have an electronic structure very similar to that of the deprotonated curcumin anion, with one bound electronically excited state and two low-lying anion resonances. Furthermore, all of the deprotonated curcuminoid anions have similar adiabatic detachment energies and show clear evidence of internal conversion following photoexcitation. Therefore, the curcuminoids studied here may be expected to perform a similar biological and/or therapeutic role to curcumin, given their similar electronic structure and energy transfer dynamics.

## Graphical abstract

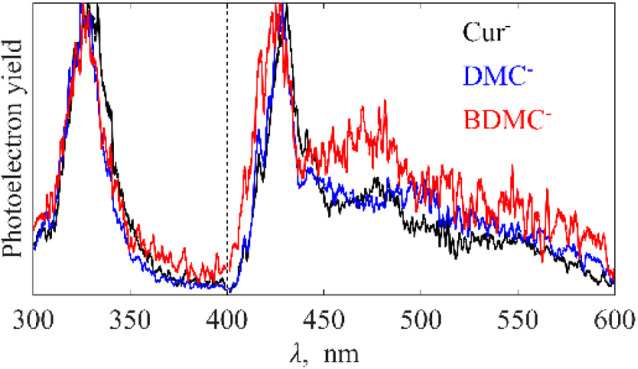

## Introduction

The spice, turmeric, has well-documented antioxidant, anti-inflammatory, antimicrobial, and anticancer properties, which have often been attributed to the curcuminoids.^1–4^ These polyphenol compounds include curcumin, the principal and best-studied chromophore in turmeric, as well as demethoxycurcumin and bisdemethoxycurcumin, which have one or two fewer terminal methoxy groups than curcumin, and are shown in Figure 1.^5^ There is significant effort being undertaken to harness the biological activity of turmeric to utilize as a therapeutic.^5–8^ First, this leads to an interest in which component species of turmeric are biologically active: curcumin only, the curcuminoids more broadly, or indeed another molecular component of turmeric altogether. Second, assuming that curcumin is biologically active, researchers are also trying to overcome its limited bioavailability, which limits its application as a potential drug.^9^ This has led to an interest in demethoxycurcumin and bisdemethoxycurcumin specifically, as their different terminal substituents have been shown to improve bioavailability compared to curcumin, as well as leading to improved stability under physiological conditions.^6,10–12^ However, it is unclear if the electronic structure of the curcuminoids is similar to curcumin, which may determine if the demethoxy variants can perform the same biological role. Furthermore, while the potential of curcumin as a photodynamic therapy agent is established, efforts are underway to investigate the potential of naturally occurring and synthetic curcuminoids, which requires an understanding of their photochemistry.^13,14^

**Figure 1:**
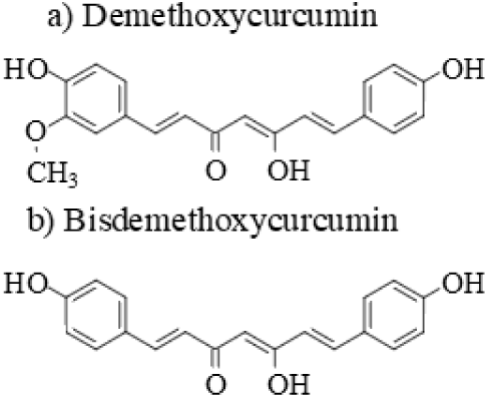
Structure of the enol tautomers of a) demethoxycurcumin and b) bisdemethoxycurcumin.

While there has been a large amount of spectroscopic research performed on the structure and dynamics of curcumin, particularly in the solution phase, there has been less work performed on the other curcuminoids.^13,15–17^ Largely, this work has demonstrated the presence of a bright electronically excited state near λ ∼ 420 nm, which gives rise to the orange/yellow color of turmeric, and additionally, it has been shown that the absorbance maximum is slightly blue-shifted with the loss of methoxy groups.^15^ However, much of the previous spectroscopic work on curcuminoids, which has predominantly been performed in the solution phase, is challenging to interpret. While chromatography can help, it is often difficult to prepare pure samples of a specific curcuminoid, as commercial starting materials contain curcumin, demethoxycurcumin, and bisdemethoxycurcumin. Furthermore, there is evidence that curcuminoids adopt numerous charge states at physiological pHs and that numerous conformers, including enol-keto tautomers, can be present, as well as distinct deprotonated forms for anionic species.^18,19^ To overcome the challenges of solution-phase spectroscopy, here we use mass-selected gas-phase ion spectroscopy to isolate the deprotonated anions of demethoxycurcumin (DMC^–^) and bisdemethoxycurcumin (BDMC^–^) and interrogate their electronic structure independently. Furthermore, assuming that the pH-dependent behavior of the curcuminoids is comparable to curcumin, it is likely that DMC^–^ and BDMC^–^ are present under biological conditions, such that we are directly studying biologically relevant species.^20^ While there has been some mass spectrometry performed on the curcuminoids,^19,21^ there has been no gas-phase spectroscopic work to study the electronic structure of DMC^–^ or BDMC^–^.

Recently, we applied the same approach (photoelectron imaging, electron action spectroscopy, and electronic structure calculations) to study the electronic structure of the deprotonated curcumin anion (Cur^–^), which was the first time gas-phase spectroscopy had been performed on any curcuminoid.^22^ While some evidence has been observed for the diketo form of the curcuminoids, it has been shown that the keto–enol form (called enol hereafter and shown in Figure 1) is typically the ground state.^23–26^ Our recent experiments indicated that only the lowest energy form of Cur^–^ was present in the gas phase, which electronic structure calculations identified as an enol form with an intramolecular hydrogen bond which was deprotonated on the terminal phenol group.^22^ Electron loss from Cur^–^ was seen via direct detachment, from which the EA of Cur (deprotonated neutral, arising from electron loss from Cur^–^) was determined to be 2.8 eV. Additionally, thermionic emission, where internally hot, ground-state anions boil off electrons statistically, was observed at all photon energies.^27,28^ Three bright electronically excited states were observed, one bound and two as resonances in the detachment continuum, which mediated the thermionic emission. Evidence was also seen for a fragmentation pathway, which resulted in the formation of a carbanion, tentatively identified as C_6_H_3_(OH)­(OCH_3_)­CHCH^–^ with mass-to-charge ratio *m*/*z* = 149 amu, by electronic structure calculations. As the dissociation process was determined to be a multiple-photon process, it was therefore unlikely to occur in the solution phase or under biological conditions. The observed photodetachment processes of Cur^–^ are summarized in Figure 2. Fundamentally, the work demonstrated the intrinsic photostability of Cur^–^, as all destructive pathways were high-lying energetically. While the cross-section for photoexcitation was large for all the excited states, we also observed internal conversion (IC) back to the ground state (through observation of thermionic emission and statistical fragmentation), which would likely allow the internal excitation to be quenched via interactions with the solvent in biological settings.

**Figure 2:**
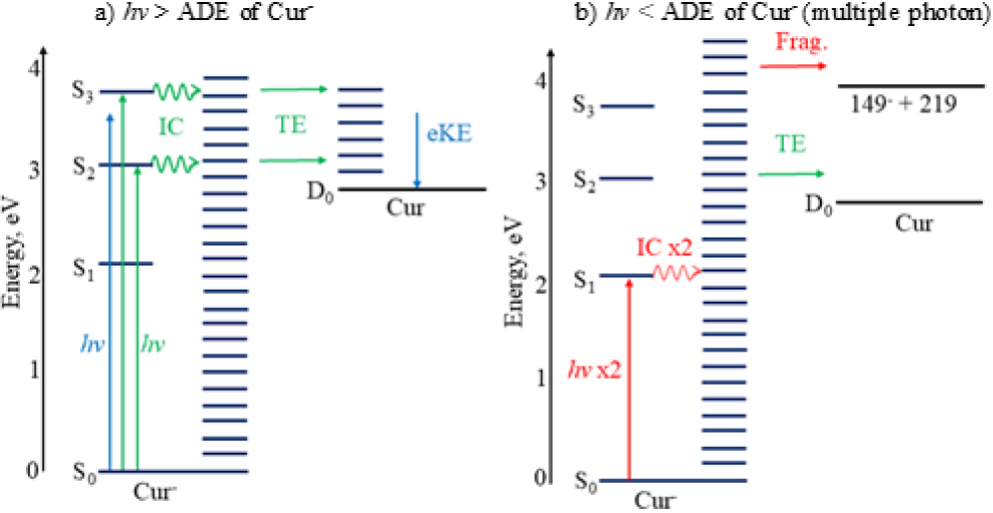
Summary of the electron loss processes observed for Cur^–^ at photon energies a) above (one-photon direct detachment and thermionic emission (TE), via internal conversion (IC)) and b) below (multiple-photon thermionic emission and photodissociation followed by photodetachment of the anionic fragment (Frag.)) the adiabatic detachment energy.

Here, we isolate DMC^–^ and BDMC^–^ in the gas phase and study their electronic and nuclear structure using photoelectron imaging, electron action spectroscopy, and electronic structure calculations. By comparing the new results for DMC^–^ and BDMC^–^ to each other, and the previous work on Cur^–^, we determine that the loss of methoxy groups has little effect on the electronic structure or photochemistry of the curcuminoids.

## Methods

The ion spectrometer used in this work has been described in detail elsewhere and will only be briefly summarized here.^29,30^ Electrospray ionization of a 10 mM solution of curcumin dissolved in a basic solution of methanol produced DMC^–^ and BDMC^–^, in addition to Cur^–^. Sufficient base was added to the curcumin solution to induce a color change from yellow to orange/red (pH ∼ 8), which has been attributed to the presence of anionic forms.^20^ It is unlikely that we will produce radical anions of the curcuminoids (i.e., without deprotonation), given that no curcuminoid ion signal was observed without the addition of base. The anions pass into a vacuum through a capillary and are guided and trapped using radiofrequency fields. The different deprotonated curcuminoid anions are separated by their time-of-flight using a Wiley–McLaren mass spectrometer (Cur^–^ 367 amu, DMC^–^ 337 amu, and BDMC^–^ 307 amu).^31^ The mass-selected anions of interest are overlapped with a nanosecond laser, which is either the second or third harmonic of a Nd:YAG (photon energy *hν* = 3.49 or 2.33 eV) or the tunable output of a *hν* = 3.49 eV pumped optical parametric oscillator. Photoelectrons produced are velocity map imaged onto a position-sensitive detector, consisting of microchannel plates and a phosphor screen. The resulting image is processed via the polar onion peeling (POP) algorithm and contains the photoelectron spectrum on an electron kinetic energy (eKE) scale, as well as the photoelectron angular distribution (PAD), which is characterized by an anisotropy parameter −1 < β_2_ < 2.^32,33^ Electron action spectroscopy is performed by recording the intensity of the photoelectron signal as a function of wavelength.^34^

Electronic structure calculations are utilized to determine the optimized geometries and energetics of the curcuminoids, as well as potential photoproducts. Geometries were optimized and confirmed using vibrational analysis, and all energies (except vertical excitation energies (VEE)) were zero-point energy corrected. Ground-state computations used the B3LYP level of theory, while the excited-state computations used time-dependent density functional theory (TD-DFT) with the Tamm–Dancoff approximation.^35,36^ All computations utilized the aug-cc-pVTZ basis set and the Gaussian 16 suite of programs.^37,38^ The same computational approach was benchmarked against other computational methods (CAM-B3LYP, 6-311++G­(d,p) and cc-pVTZ) by investigating the structure and energetics of Cur^–^.^22^ All of the computational approaches reported similar results, but the best agreement with the experiment was found with B3LYP/aug-cc-pVTZ, suggesting that this methodology is the best choice for the study of the curcuminoids.

## Results

### Electronic Structure Calculations of the Curcuminoids

Electronic structure calculations are used here to provide an initial view of the electronic structure of the curcuminoids, DMC^–^, and BDMC^–^, while also providing a summary of the previously reported electronic structure of Cur^–^.^22^ Computations indicated that the lowest energy isomers of DMC^–^ and BDMC^–^ are enols, with the structures shown in Figure 3. The central conjugated enol motif is stabilized by an intramolecular hydrogen bond, and the formal site of the negative charge is a deprotonated terminal hydroxyl group. A similar H-bond-stabilized enol structure was found to be the ground-state conformer of Cur^–^.^22^ For DMC^–^, there are two relatively low-lying isomers: the ground state has the negative charge localized on a phenolate group, whereas deprotonation of the hydroxyl group neighboring the methoxy group results in an isomer +0.13 eV higher in energy with methoxyphenolate character. Following collisions with room-temperature He in the trap, the ion temperature is likely to be *T* ∼ 300 K, such that thermal population of the higher-energy isomer would be unlikely (*k*_B_ *T* equivalent, *T* ∼ 1500 K). On this basis, it is most likely that a single isomer of both BDMC^–^ and DMC^–^ is present in the ion beam. The relative energetics of other DMC^–^ and BDMC^–^ isomers, including electron affinities (EA) and vertical detachment energies (VDE), are reported in Table S1 and S2 of the Supporting Information (SI) respectively.

**Figure 3:**
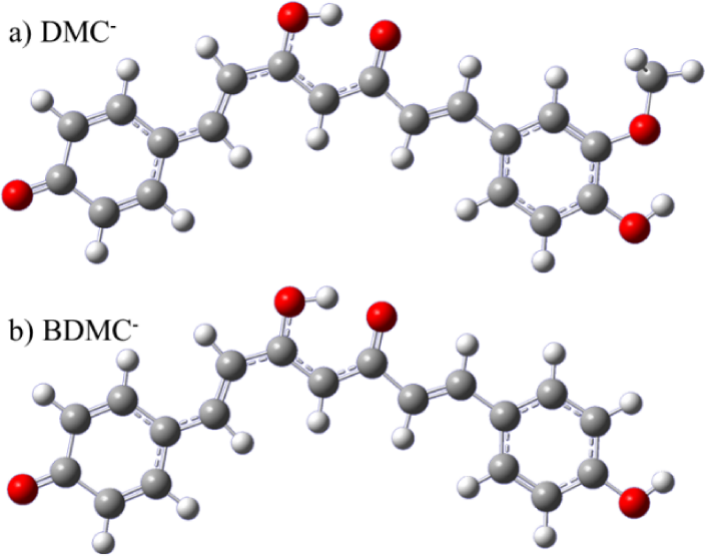
Optimized ground-state geometries of a) DMC^–^ and b) BDMC^–^. Both anions, like Cur^–^, adopt an enol configuration, stabilized by an intramolecular hydrogen bond, and are deprotonated on a terminal phenol group.

The computed electron affinities (EA), vertical detachment energies (VDE), and vertical excitation energies (VEE) for the first three electronically excited states of DMC^–^ and BDMC^–^ are shown in Table 1. Additionally, the previously reported computed and experimental values for Cur^–^ are included for comparison.^22^ The computed EAs for DMC^–^ (2.95 eV) and BDMC^–^ (2.97 eV) are similar to each other and slightly higher than the experimentally observed adiabatic detachment energy (ADE) of Cur^–^ (2.8 eV). The slight increase in EA with the removal of a methoxy group is likely the result of destabilization of the resulting radical. In all cases, the VDEs are slightly higher than the EA, indicating that there is likely to be vibrational excitation imparted to the radical upon photodetachment, resulting from a change in geometry between the anion and the neutral, as was observed for Cur^–^.^22^

**Table 1:** Calculated Relative Energetics for the Curcuminoids, Including Electron Affinity (EA), Vertical Detachment Energy (VDE), and Vertical Excitation Energies (VEE) for the First Three Excited States^a^ ^b^

Molecule	EA, eV	VDE	VEE(S_1_)	VEE(S_2_)	VEE(S_3_)
Cur^–^	2.79	2.91	2.18	3.08	3.77
	(2.8)	(3.1)	(<2.8)	(2.88)	(3.75)
DMC^–^	2.95	3.05	2.25	3.18	3.74
BDMC^–^	2.97	3.07	2.24	3.18	3.75

a Experimental values are included in brackets for Cur^–^ from the previous work.^22^

b All energies are in eV.

Excited state calculations indicated three bright electronically excited states with some shape resonance character over the range of photon energies studied (up to 4.13 eV), with similar VEEs for all of the curcuminoids. Based on the computed EAs, we anticipate that DMC^–^ and BDMC^–^ will have a bound electronic state (S_1_) and two resonances in the detachment continuum (S_2_ and S_3_), as was previously reported for Cur^–^.^22^ Small blue shifts are predicted in the VEE for S_1_ and S_2_ with the removal of a methoxy group from the curcuminoids. In short, the computations predict that the electronic and nuclear structures of DMC^–^, BDMC^–^, and Cur^–^ are very similar, and thus we expect that the photoelectron and electron action spectra of all three curcuminoids will be similar.

### Photoelectron Imaging of DMC^–^

Photoelectron spectra of DMC^–^ recorded at various photon energies (*hν*) are shown in Figure 4, with three distinct spectral features associated with different electron loss pathways. First, a broad band, with a fixed eBE at 2.85 eV (Figure 2, blue), second a feature starting at a fixed eBE ∼1.1 eV present only at the longest wavelengths studied (Figure 4, red); and finally, a high-intensity feature at eKE = 0 (eBE ∼ *hν*) present at all *hν* but with different relative intensities (Figure 4, green). Similar features were observed in the previously reported photoelectron spectra of Cur^–^.^22^

**Figure 4:**
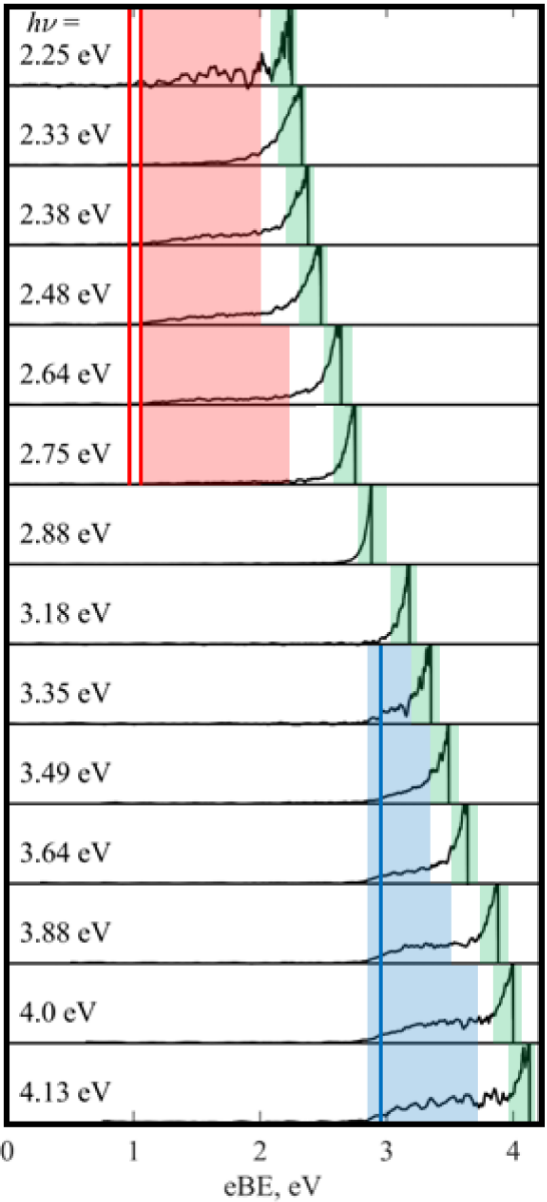
Photoelectron spectra of DMC^–^ recorded at a range of photon energies (*h*ν) in the UV and visible. The three different electron loss channels: high adiabatic detachment energy (ADE) direct detachment (blue), low ADE direct detachment (red), and thermionic emission (green) are highlighted. The calculated electron affinity for DMC (blue line) and its likely photoproducts (red lines) is also shown.

The spectral feature associated with the highest binding energy electrons occurs at a fixed eBE and is only present at photon energies *hν* > 3 eV (blue, Figure 4). Such features are usually direct detachment channels of the parent species, in this case, DMC^–^. From this, an adiabatic detachment energy (ADE) of 2.85 eV and a vertical detachment energy (VDE) of 3.25 eV can be extracted for DMC^–^, which match reasonably well with the computed EA (2.95 eV, blue line, Figure 4) and VDE (3.05 eV) for DMC^–^ (see Table 1). The spectral feature is broad, indicating a large geometry change between the anion and neutral upon photodetachment, resulting in a wide Franck–Condon envelope. The PAD for this direct detachment feature is characterized by an anisotropy parameter β_2_ ∼−0.1 ± 0.3.

At longer wavelengths (*hν* < 2.75 eV), another fixed eBE feature, likely attributable to direct detachment, is observed (Figure 4, red). However, direct detachment of DMC^–^ cannot occur at these wavelengths as *hν* is smaller than the ADE of DMC^–^. This below-threshold direct detachment spectral feature is characterized by ADE = 1.1 eV and VDE = 1.5 eV, which are much lower than those for DMC^–^ itself. Therefore, this spectral feature may arise from an isomer of DMC^–^ present in the anion beam or from direct detachment of a photoproduct of DMC^–^ via a multiple-photon process. The latter is most likely for several reasons. First, this spectral feature is observed for a narrow range of *hν* (2.25 eV < *hν* < 2.8 eV), and it switches on near the computed VEE­(S_1_) (*hν* = 2.25 eV), pointing toward a photoproduct produced via an excited-state process. Second, the computed EAs (>2.7 eV) of the isomers of DMC^–^ (Table S1) are much higher than the observed ADE (∼1.1 eV), ruling out isomers of DMC^–^ either present in the ion beam (which would also be unlikely from a thermal perspective) or produced via photoisomerization. The radical doublet anion (i.e., DMC^–^ without deprotonation) is expected to have a lower ADE than DMC^–^ (cf. nondeprotonated curcumin has an EA ∼1 eV), but this anion would be present at all wavelengths, and we are likely able to temporally separate the radical anion from the deprotonated anion DMC^–^ as a result of their different *m*/*z* (mass resolution of the spectrometer is better than $\frac{M}{\Delta M}>200$ ).^39^ It should be noted that two-photon direct detachment of the parent curcuminoid anion is ruled out, as in this case the direct detachment band would move on both the eBE and eKE axes shown in Figure 4 with changing *hν*, as eKE + eBE = 2*hν* for a concerted two-photon photodetachment.^40^ Therefore, the most likely assignment to the red spectral feature in Figure 4 is that absorption of a photon leads to photodissociation of DMC^–^ to produce an anionic fragment, which is subsequently photodetached with a second photon over the duration of the laser pulse (∼5 ns). The broadness of the spectral band indicates that the fragment undergoes a large geometry change from the anion to the neutral upon electron loss. Furthermore, we attempted to record the fragment mass spectra using a reflectron for Cur^–^ but were unsuccessful, likely because of the statistical nature of the dissociation, which would be expected to result in a broad range of arrival times for the small number of fragment ions.^22^ The identity of this fragment will be investigated in the following section using electronic structure computations.

At all *hν* studied, there is an intense feature of electrons with eKE near 0 eV (i.e., shifting on an eBE scale), which has an isotropic PAD (Figure 4, green). This spectral feature is consistent with electrons being ejected via thermionic emission, whereby a hot ground-state anion “boils off” electrons. Effectively, at near-resonant wavelengths, the anion would be photoexcited to an electronically excited state, followed by IC to the ground state to produce a vibrationally hot anion. This process may be repeated until the anion has enough internal energy to lose an electron. Given that thermionic emission is seen at all wavelengths studied, if this is an excited-state process, it is likely to be mediated by multiple excited states, as is consistent with the three electronically excited states predicted by the electronic structure calculations (Table 1). The relative intensity of this feature changes with photon energy but clearly peaks in the *hν* = 2.88 eV spectrum, and again near *hν* = 3.64 eV, pointing toward an excited state near λ ∼ 430 and 340 nm, close to the computed VEE for S_2_ and S_3_.

### Photoelectron Imaging of BDMC^–^

Figure 5 shows the photoelectron spectra of BDMC^–^ recorded at multiple *hν* in the visible and UV. The photoelectron spectra are very similar to those recorded for DMC^–^ and are shown in Figure 4. Again, there are three spectral features: direct detachment from both BDMC^–^ (blue, Figure 5) and a photofragment (red, Figure 5), as well as thermionic emission (green, Figure 5). The justification for these assignments is the same as in the photoelectron spectra of DMC^–^, as described in the previous section. The PAD for the direct detachment feature is characterized by β_2_ ∼ 0 ± 0.5. The ion signal was lower for BDMC^–^ than DMC^–^, resulting in a lower signal-to-noise ratio in the photoelectron spectra and therefore greater uncertainty in the β_2_ value. At certain wavelengths with low laser power (e.g., *hν* = 3.35 eV), it was not possible to record a BDMC^–^ photoelectron spectrum of sufficient quality to be reported.

**Figure 5:**
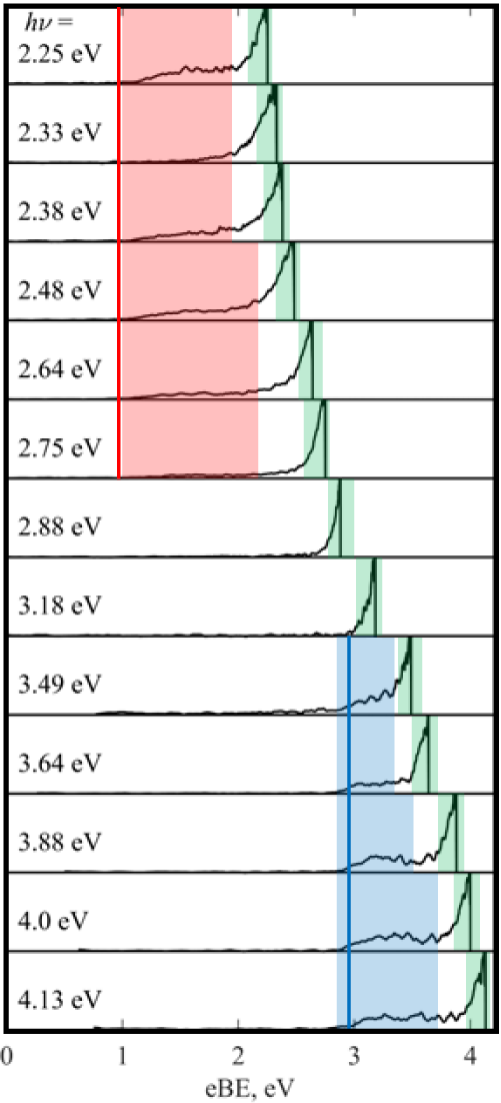
Photoelectron spectra of BDMC^–^ recorded at a range of photon energies, *h*ν. The three electron loss pathways are highlighted: high adiabatic detachment energy (ADE) direct detachment (blue), low ADE direct detachment (red), and thermionic emission (green). The calculated electron affinity (EA) of BDMC (blue line) and its likely photoproducts, 119^–^ (red line), are also shown.

**Figure 6:**
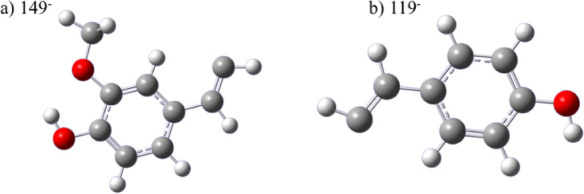
Optimized structures of the most likely fragments a) 149^–^ and b) 119^–^. Photodissociation of Cur^–^ would produce 149^–^, photodissociation of BDMC^–^ would produce 119^–^, while photodissociation of DMC^–^ could produce either 149^–^ or 119^–^.

The photoelectron spectra of BDMC^–^ yield an experimental ADE = 2.9 eV and experimental VDE = 3.3 eV (blue, Figure 5), which match reasonably well with the computed EA (2.97 eV, blue line, Figure 5) and VDE (3.07 eV) shown in Table 1. Again, a multiple-photon photodissociation and photodetachment channel is observed at *hν* < ADE (red, Figure 5), and for BDMC^–^, the corresponding fragment has an ADE = 1.1 eV and a VDE = 1.5 eV. Similar to DMC^–^, thermionic emission peaks in the *hν* = 2.88 eV spectrum, indicating that there is a nearby excited state, with another potentially present in the 3.64 eV spectrum, where there is an increase in the relative intensity of thermionic emission (green, Figure 5). By comparison to the computed VEEs (Table 1), the excited states mediating thermionic emission above the threshold are likely to be S_2_ and S_3_.

### Electronic Structure Calculations of the Fragments

The photoelectron spectra of DMC^–^ and BDMC^–^ both have spectral features arising from the direct detachment of anionic fragments, produced by photodissociation of the parent anions (red, Figures 4 and 5). As secondary mass spectrometry was unsuccessful in determining the fragment mass, we attempted an assignment by computing the electronic structure of numerous likely fragments suggested by mass spectrometry studies.^19,21,24,41,42^ Both fragments have ADEs of approximately 1.1 eV, suggesting a carbon-localized anion, as typically oxygen-localized species would have a larger ADE (e.g., EA­(OMe) = 1.57 eV, EA­(OH) = 1.82 eV, and EA­(O) = 1.44 eV).^43–45^ The EA and VDE for all of the potential fragments studied are reported in Table S3, but the most likely assignment is C_6_H_3_(OH)­(OMe)­CHCH^–^ (149^–^) and the demethoxy version C_6_H_4_(OH)­CHCH^–^ (119^–^), as the computed values (red line in Figures 4 and 5) match well with the experimental spectral features (red in Figures 4 and 5). The optimised geometries of 149^–^ and 119^–^ are shown in Figure 6. While only 119^–^ can be formed from BDMC^–^, both fragments may be produced from DMC^–^. It is therefore challenging to determine whether 119^–^ or 149^–^ is the most likely photoproduct of DMC^–^, as the computed EA and VDE for both fragments are very similar. However, previous mass spectrometry of DMC^–^, which induced fragmentation via collisions rather than light, reported a preference for the formation of 149^–^ rather than 119^–^.^41^ The bond dissociation energies (*D*_0_) for the asymptotes of the most likely anionic fragments are computed and shown in Table 3, but for all pathways *D*_0_ ∼ 4 eV, indicating that it will be a multiple-photon process at the wavelengths of interest (*hν* < 2.75 eV). The VEE of the first excited state of both fragments is also computed and shown in Table 2, indicating that both fragments have electronically excited states in the multiple-photon region (i.e., below threshold).

**Table 2:** Electron Affinities (EA) of the Corresponding Neutral, Vertical Detachment Energies (VDE), and Vertical Excitation Energy (VEE) of the Most Likely Photofragments of BDMC^–^, DMC^–^, and Cur^–^: 149^–^ and 119^–^ ^a^ ^b^

Fragment	EA	VDE	VEE(S_1_)
C_6_H_3_(OH)(OCH_3_)CHCH^–^ (149 amu, 149^–^)	1.04 (1.1)	1.57 (1.5)	2.36
C_6_H_4_(OH)CHCH^–^ (119 amu, 119^–^)	0.98	1.51	2.56

a Experimental values of the EA and VDE are included in parentheses for 149^–^, which was previously observed as the photoproduct of Cur^–^.

b All energies are in eV.

### Electron Action Spectroscopy of the Curcuminoids

Electron action spectroscopy can act as a gas-phase analogue to absorption spectroscopy, where peaks in the electron yield indicate the location of electronically excited states, assuming that photoexcitation to an anionic electronically excited state results in electron loss.^34,46,47^ The electron action spectra for DMC^–^ and BDMC^–^ are shown in Figure 7, alongside the previously reported spectrum for Cur^–^.^22^ While it would be desirable to normalize the electron action spectra to account for fluctuations in laser power (*P*), it is not possible as the spectra contain contributions from one-, two-, and three-photon electron loss processes, which would require distinct normalizations, e.g., ∝ *P*, *P* ^2^, and *P* ^3^. However, the fluctuation in laser power as a function of wavelength is plotted (Figure 7, blue dashed line) to demonstrate that the structure of the action spectra reports changes in the electronic structure of the curcuminoids. Furthermore, the above-threshold action spectra for the curcuminoid anions, i.e., *hν* > ADE, where one-photon processes are expected to dominate, have been normalized to account for the number of photons and are shown in Figure S1 (SI). The general structure of all three action spectra is very similar with two sharp bands near λ ∼ 330 and 430 nm (green, Figure 7), while at longer wavelengths, electron loss still occurs, but the electron yield is lower, and the structure is different for each of the anions (red, Figure 7). The computed VEE for the S_1_, S_2_ and S_3_ states of all the curcuminoids (red arrows), and the recorded ADE for each anion (red line) are shown.

**Figure 7:**
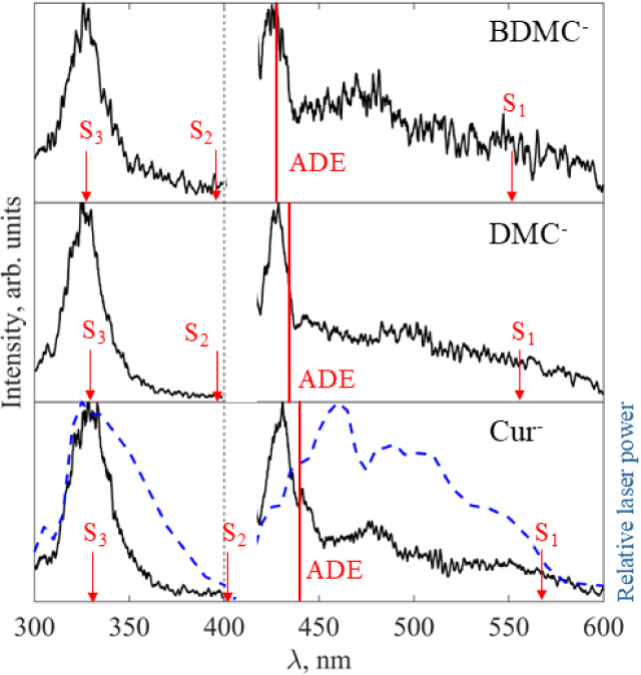
Electron action spectra of the curcuminoids: Cur^–^, DMC^–^ and BDMC^–^, with the normalized relative laser power plotted on the Cur^–^ action spectrum (dashed, blue line). The ADE for each anion is denoted by the vertical red line, to separate the one-photon region (*h*ν > ADE) and the multiple-photon region (*h*ν < ADE) of the electron action spectra. Peaks in the action spectra are likely to arise from the presence of an electronically excited state, and so the computed vertical excitation energies (VEE) for the S_1_, S_2_, and S_3_ excited states of each anion are indicated with red arrows. The electron action spectra in the region between λ = 400–420 nm is not shown, due to very low laser power.

The peaks near λ ∼ 330 and 430 nm are in the one-photon region of the spectrum (*hν* > ADE) for all of the curcuminoids and are close to the computed VEE for S_2_ and S_3_ (Table 1). From the photoelectron spectra recorded near these peak wavelengths, we see both direct detachment of the parent (blue, Figures 4 and 5) and an increased propensity for thermionic emission (green, Figures 4 and 5). There is a good match between the computed VEE­(S_3_) and the peak in the experimental spectra near λ ∼ 330 nm, and while the 300–320 nm region of the spectra mirrors the laser power curve, the region from 350–330 nm is different, and its structure cannot be explained by considering the direct photodetachment cross-section, providing evidence for an electronically excited state and supporting an assignment of VEE­(S_3_) ∼3.75 eV for Cur^–^, DMC^–^, and BDMC^–^. While there is a larger discrepancy between the computed VEE­(S_2_) and the peak of the action spectra (near λ ∼ 430 nm) for all of the curcuminoids, the very low laser power at 400 nm < λ < 420 nm means we cannot record electron action spectra in this region, and therefore, it is likely that VEE­(S_2_) ∼ 2.85–3.0 eV for all the curcuminoids. From the electron action spectra, we see that there is a small shift to higher VEE­(S_2_) for DMC^–^ and BDMC^–^ compared to Cur^–^, while VEE­(S_3_) is largely unchanged. This was predicted by electronic structure computations (Table 1).

Additionally, electron loss is observed for 450 nm < λ < 600 nm for all the curcuminoids, and given that these wavelengths are below threshold for all of the anions studied (*hν* < ADE), in this region (red, Figure 7), only multiple photon processes can occur. In the photoelectron spectra at these wavelengths, we observe thermionic emission (green, Figures 4 and 5) along with photodissociation and subsequent photodetachment (red, Figures 4 and 5). The onset of electron loss is near λ ∼ 600 nm for all of the curcuminoids, which is close to the computed VEE­(S_1_), indicating that these multiple photon electron loss processes are being mediated by S_1_. The onset for electron loss is gradual, which may reflect the contribution of hot bands, particularly as, in this region, internally hot anions (parents and fragments) are produced via sequential absorption of multiple photons and IC. Furthermore, the computed VEE­(S_1_) for the most likely fragments, 119^–^ and 149^–^, are also in the multiple photon region, where the fragment signal is observed in the photoelectron spectra (Figures 1 and 2), potentially enhancing the propensity for this multiple photon process (red, Figures 4 and 5). The structure of the electron action spectra in this region (red, Figure 7) is complex and different for each of Cur^–^, DMC^–^, and BDMC^–^. It is likely complex because distinct multiple photon multistep processes are competing, where each step of each process has distinct wavelength-dependent cross sections. The difference between the electron action spectra of the different curcuminoids likely reflects different branching ratios between fragmentation and thermionic emission in different curcuminoids, as well as different dissociation asymptotes being preferred for different curcuminoids and different photodetachment cross sections for different photofragments.

## Discussion

For comparison, the photoelectron spectra of all three curcuminoids recorded at three photon energies are shown in Figure 8. The spectrum recorded at the highest photon energy (*hν* = 3.88 eV, Figure 8a) shows that there is a small increase in both ADE and VDE with the removal of a methoxy group (∼0.1 eV). This effect is captured in the electronic structure calculations (Table 1), where the computed EAs also accurately reproduce the experimental ADEs. Given the electron-donating properties of the methoxy group via resonance structures, this trend is in line with previous observations, including, for example, that the addition of a different electron-donating group, in this case the methyl group, reduced the EA of phenoxy.^48^ However, this effect may be small given that oxygen is electronegative and electron-withdrawing via induction, like fluorine, which previous work has indicated increases the EA of phenoxy when it is added.^49^ All of the direct detachment bands are broad, indicating a significant geometry change upon photodetachment, and while a substantial difference in EA and VDE is predicted for all the anions using electronic structure computations, this effect is underestimated (Table 1). One explanation is that autodetachment from S_2_ and S_3_ overlaps with the direct detachment band (blue, Figures 4 and 5) leading to broadening, and this may explain the discrepancy between the computed and experimental VDE for all of the curcuminoids.^50^ Given that we observe thermionic emission in all of the photoelectron spectra, we may also expect autodetachment to be present at all *hν* > VEE­(S_2_).

**Figure 8:**
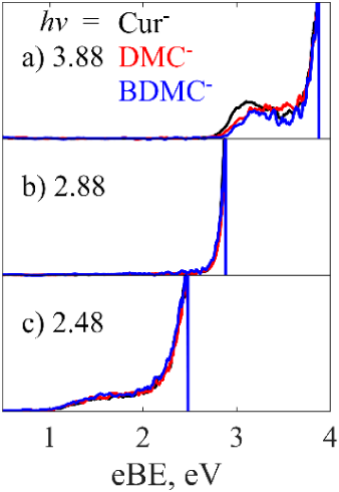
Comparison of the photoelectron spectra of Cur^–^ (black), DMC^–^ (red), and BDMC^–^ (blue) at a) *h*ν = 3.88 (one-photon direct detachment and thermionic emission), b) 2.88 (near-threshold thermionic emission), and c) 2.48 eV (multiple-photon fragmentation and thermionic emission).

The relative intensity of direct detachment and thermionic emission is also different for the different curcuminoids at *hν* = 3.88 eV (Figure 8), with an increased preference for thermionic emission for BDMC^–^ compared to that of DMC^–^, and DMC^–^ compared to that of Cur^–^. The propensity for direct detachment is controlled in part by the Franck–Condon envelope, which given that *hν* ≫ ADE, is expected to be fully accessible for all the curcuminoids. There is not a significant difference in the VEE­(S_3_) between the curcuminoids, such that photoexcitation at *hν* = 3.88 eV is likely to be as close to resonance for each of the curcuminoids. Therefore, the discrepancy may indicate a small but fundamental increase in the photoexcitation cross-section compared to the photodetachment cross-section for curcuminoids with fewer terminal methoxy groups. Alternatively, if the photoexcitation and photodetachment cross-sections are similar for all the curcuminoids, then the increased proportion of thermionic emission (BDMC^–^ > DMC^–^ > Cur^–^) may reflect an increased efficiency for IC compared to autodetachment following photoexcitation.

The computational and experimental results indicate that BDMC^–^, DMC^–^, and Cur^–^ have a bound S_1_ state, as well as two energetically accessible and optically bright S_2_ and S_3_ resonances in the detachment continuum, with similar VEE­(S_*n*_) (*n* = 1, 2, or 3) for all of the curcuminoids. It is challenging to accurately determine the location of the S_1_ state for the deprotonated curcuminoid anions, given that it requires multiple photons to lose an electron in this region (red, Figure 7), but it is likely that VEE­(S_1_) ∼ 2.1 eV (λ ∼ 600 nm), where electron loss turns on for all the anions studied. The computations (Table 1) predict a small increase in VEE­(S_1_) for DMC^–^ (2.23 eV) and BDMC^–^ (2.27 eV), compared to Cur^–^ (2.18 eV), but this difference is difficult to quantify experimentally given the complex structure in the multiple-photon region of the electron action spectra (Figure 7). The removal of a methoxy group from curcumin appears to blue-shift the experimental VEE­(S_2_) slightly (<5 nm) in an effect which is largely captured by the electronic structure calculations (Table 1). However, there is a discrepancy between the computed and experimental VEE­(S_2_)­s, although this is most likely to result from very low laser powers near λ ∼ 400 nm. Furthermore, a similar blue-shift in the absorption maxima of the solution-phase absorption spectra of the curcuminoids in ethanol (Cur λ_max_ = 429 nm, DMC λ_max_ = 424 nm, and BDMC λ_max_ = 419 nm), where neutral forms are expected to dominate, was previously reported, and this is likely to correspond to excitation to S_2_.^15^ In contrast, VEE­(S_3_) appears to be very similar for all of the curcuminoids, and the experiments and computations are in excellent agreement. Given the prevalence of thermionic emission at all *hν* studied, as well as the presence of photofragments produced via a statistical process, it is highly likely that all the curcuminoids undergo relatively rapid IC following photoexcitation (compared to the lifetime of the excited state), as was previously reported for Cur^–^ and other anions.^22,51^

In the lowest energy photoelectron spectrum recorded at *hν* = 2.48 eV (Figure 8c), there is evidence for multiple-photon photodissociation and subsequent photodetachment of the anionic fragment (Figure 8), as well as thermionic emission. Both electron loss and photofragmentation, based on the computed *D*_0_s (Table 3), are two-photon processes at ADE < λ < 600 nm. As there is competition between thermionic emission and photodissociation for all of the curcuminoids at longer wavelengths, with both processes switching on at *hν* ∼ VEE­(S_1_), it is probable that both electron loss and dissociation are statistical processes mediated by S_1_. Effectively, photoexcitation to S_1_ is followed via IC to S_0_, in a repeating cycle (two or more times), until the curcuminoid anion has enough energy to fragment or lose an electron statistically. This is shown for Cur^–^ in Figure 2b. To test for the presence of multiple-photon processes, laser fluence dependence measurements were attempted on Cur^–^ but were unsuccessful, as the reduced laser power meant data of sufficient quality (e.g., acceptable signal-to-noise ratio) could not be recorded.^22^ Given the reduced ion intensity of DMC^–^ and BDMC^–^ compared to Cur^–^, we therefore did not attempt additional fluence-dependent measurements for the curcuminoids. Furthermore, previous work has demonstrated that fluence-dependent measurements can be inconclusive in determining the presence of multiple-photon processes, as each distinct step (e.g. photoexcitation, photodissociation, orphotodetachment) is governed by adifferent cross section.^52^

**Table 3:** Computed Bond Dissociation Energies (*D*_0_) of the Deprotonated Anionic Curcuminoids (DMC^–^, BDMC^–^, and Cur^–^) Resulting in the Most Likely Product Channels (Involving 149^–^ or 119^–^, Where 218 and 188 Denote the Mass of the Neutral Cofragments)^a^

Deprotonated curcuminoid anion	D_0_(C–C) (channel)
Cur^–^	3.85 (149^–^ + 218)
DMC^–^	4.16 (119^–^ + 218)
	4.10 (149^–^ + 188)
BDMC^–^	4.17 (119^–^ + 188)

a All energies are in eV.

The observed photodetachment dynamics of DMC^–^ and BDMC^–^ are very similar to each other and to the previously studied Cur^–^.^22^ While it should be noted that ion spectroscopy only directly probes the high-lying molecular orbitals of an anion, as well as the resulting neutral states accessed via electron removal from these high-lying MOs (Koopmans’ theorem), these are typically the orbitals which are most affected by substitution, as well as the orbitals which play a key role in chemical or biological function, i.e., electron loss processes or fragmentation. Therefore, the results presented here indicate that all the curcuminoids have very similar electronic structures and that the structure and dynamics of DMC^–^ and BDMC^–^ are also accurately described by Figure 1.

In this work, the curcuminoids have been studied while isolated as anions in the gas phase, which is far from the biological conditions where a therapeutic would be active. However, by isolating BDMC^–^, DMC^–^ and Cur^–^ in the gas phase, we have gained a clear picture of the intrinsic structure and dynamics of the curcuminoids, without complicating factors, such as the presence of many molecular species, the effect of the solvent, and many forms (e.g., isomers, tautomers, deprotomers) being present. This is significant as it allows us to consider the potential similarities between the curcuminoids under biological conditions.

While the different curcuminoids have different molecular structures and therefore may potentially have distinct chemistry (different products or reaction pathways) or binding patterns, our work indicates that the anions all adopt very similar structures (enol, planar structures with terminal deprotonation) in the gas phase. Furthermore, our work indicates that all of the deprotonated curcuminoid anions are relatively stable, with high-lying dissociation (and electron loss) pathways, such that ready fragmentation under biological conditions is unlikely for any of the species studied. The locations of the electronically excited states for all three deprotonated curcuminoid anions are very similar, indicating that the terminal methoxy groups do not impact the photochemical behavior. Furthermore, strong evidence is seen for the presence of IC following photoexcitation for BDMC^–^, DMC^–^, and Cur^–^, where the excitation of the vibrationally hot ground-state anion is likely to be quenched through collisions in the solution phase. This could provide some degree of photoprotection or even a mechanism for local heating of the solution, which has been suggested as the route for curcumin to act as a photodynamic therapy agent.^14,22^ Ultimately, our conclusion that the structure and dynamics of the deprotonated curcuminoid anions are very similar is significant because, regardless of the removal of terminal methoxy substituents, the curcuminoids are likely to perform a similar biological role to curcumin. Given previous reports of their increased bioavailability and stability, this may indicate that demethoxycurcumin and bisdemethoxycurcumin have improved potential as therapeutics over curcumin.

## Conclusions

Gas-phase ion spectroscopy of two of the deprotonated anionic forms of the most prevalent curcuminoids, demethoxycurcumin (DMC^–^) and bisdemethoxycurcumin (BDMC^–^), indicates that the spectroscopy, energetics, and dynamics of the molecules are very similar to the deprotonated anion of the primary polyphenol, curcumin (Cur^–^). Specifically, like Cur^–^, DMC^–^, and BDMC^–^ have one bound electronically excited state as well as two low-lying anion resonances, and the corresponding neutrals have similar electron affinities (EA ∼ 2.8–2.9 eV). Furthermore, the presence of thermionic emission and statistical fragmentation indicates a preference for IC following photoexcitation. Taken together, our results indicate that the terminal substituents have little impact upon the electronic structure of the curcuminoids.

## Supplementary Material



## Data Availability

The data, which support the findings reported here, are avaliable online at https://doi.org/10.5281/zenodo.18599599.

## Notes

Computed energetics of different isomers of DMC^–^ and BDMC^–^, and potential photofragments, as well as the normalized above-threshold electron action spectra for all of the deprotonated curcuminoid anions (PDF)
