# Supplementary material for: Structure and Dynamics of the Deprotonated Demethoxycurcumin and Bisdemethoxycurcumin Anions
Source: J Phys Chem A. 2026 Feb 12;130(8):1631–9. doi: 10.1021/acs.jpca.5c08039 (PMC12951561; doi:10.1021/acs.jpca.5c08039)
Supplement: Supplementary file 1 [file jp5c08039_si_001.pdf]

Supporting Information for Structure and dynamics of the Deprotonated Demethoxycurcumin and Bisdemethoxycurcumin Anions

*Jemma A. Gibbard**

Department of Chemistry, Durham University, Durham DH1 3LE, United Kingdom

Email: jemma.gibbard@durham.ac.uk

Isomer	Relative E, eV	EA, eV	VDE, eV
Enol, H bond, phenolate (Fig. 3a)<br>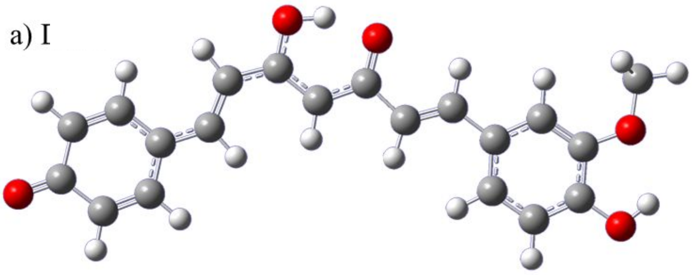	0	2.95	3.05
Enol, H bond, methoxyphenolate<br>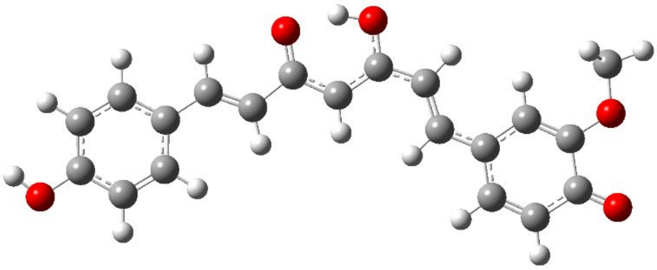	0.13	2.81	2.92
Keto, phenolate, bent<br>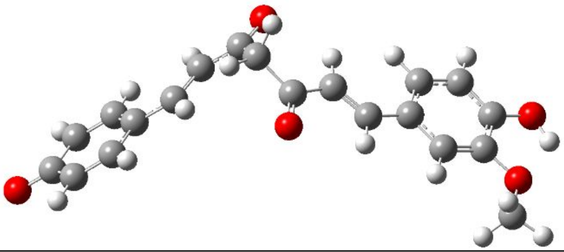	0.35	2.91	3.07
Keto, methoxyphenolate, bent<br>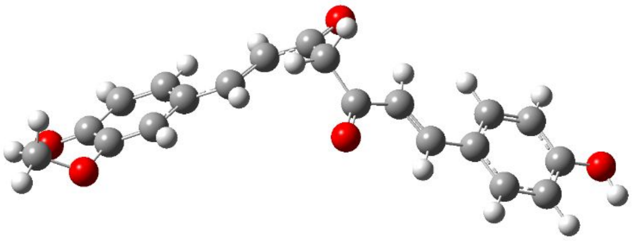	0.44	2.75	3.05

Central O^-<br>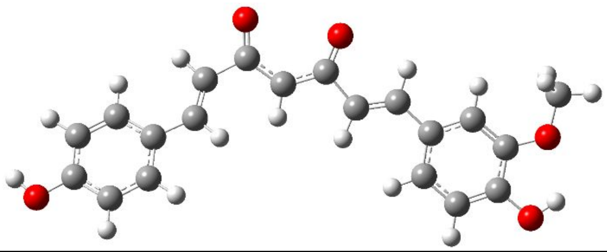	1.01	2.72	2.95

Table S1: The computed energetics of different minimum energy geometries of DMC^-. The keto or enol label refers to the characteristics of the central moiety, i.e. diketo or keto-enol, H-bond indicates the presence of an intramolecular H bond in that central keto-enol moiety, the carbon backbone of the molecule is largely planar, unless labelled bent, and terminal phenolate, terminal methoxyphenolate or central O^- indicates the location of the deprotonated alcohol group where the formal negative charge resides.

**Isomer**	**Relative E, eV**	**EA, eV**	**VDE, eV**
Enol, H bond, terminal O^- (Fig. 3b)<br>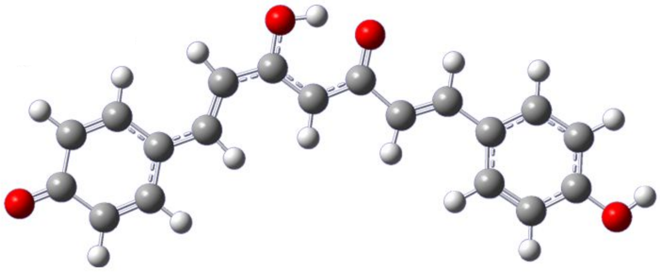	0	2.97	3.07
Keto, terminal O^-, bent<br>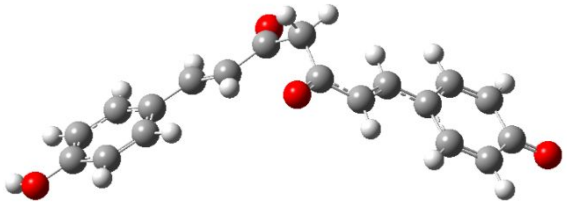	0.34	2.92	3.08
Central O^-<br>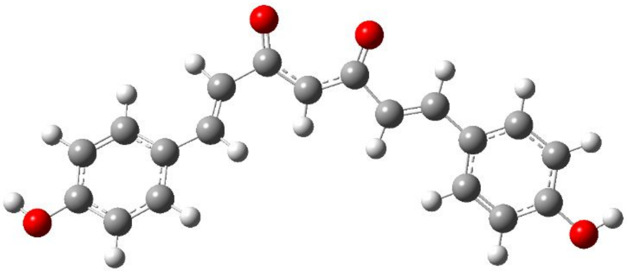	1.01	2.73	2.95

Table S2: The computed energetics of different minimum energy geometries of $BDMC^-$. The keto or enol label refers to the characteristics of the central moiety, i.e. diketo or keto-enol, H-bond indicates the presence of an intramolecular H bond in that central keto-enol moiety, the carbon backbone of the molecule is largely planar, unless labelled bent, and terminal (phenolate) or central O^- indicates the location of the deprotonated alcohol group where the formal negative charge resides.

Potential fragment of DMC⁻ or BDMC⁻	EA, eV	VDE, eV
$C_6H_4(OH)CHCH^-$ (119⁻, Fig. 6b)	0.98	1.51
$^{*-}C_6H_3(OH)(OCH_3)$ (123 a.m.u.)	0.88	1.29
$C_6H_4(O)CHCHCCH^-$ (143 a.m.u)	2.50	2.52
$C_9H_5(O)O^-$ (145 a.m.u.)	2.53	2.56
$C_6H_3(OH)(OCH_3)CHCH^-$ (149⁻, Fig. 6a)	1.04	1.57
$C_6H_3(OCH_3)(CHCH_2)O^-$ (149 a.m.u.)	2.02	2.08
$C_6O_2H_3CHCHCCH^-$ (158 a.m.u.)	2.42	2.52
$C_9H_4O_3^-$ (160 a.m.u.)	2.43	2.59
$C_6H_3(O)(OCH_3)CHCHCCH^-$ (173 a.m.u)	2.37	2.53
$C_9H_4(O)(OCH_3)O^-$ (175 a.m.u.)	2.24	2.33
$C_6H_4(CHCHCOCHCO)O^-$ (187 a.m.u.)	2.89	2.99
$C_6H_3(OCH_3)(CHCHCOCHCO)O^-$ (217 a.m.u.)	2.66	2.80

Table S3: The computed energetics of different potential anionic fragments of DMC⁻ and BDMC⁻, reported in previous gas-phase mass spectrometry studies of the curcuminoids.[1-5] In previous work fragments with masses 217, 176, 175,149 and 187 were predominantly observed for negative ion electrospray ionization mass spectrometry of DMC⁻, whilst 187, 145, 143 and 119 were observed for BDMC⁻ under analogous conditions.[1] Many of the fragment structures observed between the two curcuminoids differ by a methoxy group. When structures were reported in the previous work, these were used as starting points for geometry optimisations, and the possibility of numerous low-lying structural isomers was considered. Some low intensity unassigned fragments were observed in the mass spectrometry studies of curcuminoids, and energetics have not been computed for these species here.

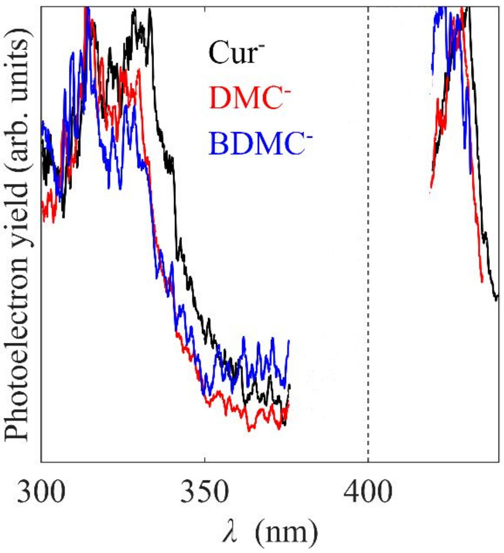

Figure S1: The electron action spectra of Cur⁻, DMC⁻ and BDMC⁻ after normalisation to account for the different number of photons at each wavelength, as a result of fluctuations in laser power. Only the above threshold, one-photon portion of the spectra can be normalised, i.e. *hv* > ADE, as below threshold multiple photon processes compete to lead to electron loss. Furthermore, around λ ~ 400 nm, the laser power is very low (< 5mW) and therefore the error bars on the power measurement are large, which can induce artifacts in the spectra. For this

reason, the normalised spectra are only reported when the laser power > 5mW. The dashed line at λ ~ 400 nm indicates where the optical parametric oscillator was reconfigured, and spectra above and below were recorded separately.

References

(1) Jiang, H.; Somogyi, Á.; Jacobsen, N. E.; Timmermann, B. N.; Gang, D. R. Analysis of curcuminoids by positive and negative electrospray ionization and tandem mass spectrometry. *Rap. Comm. Mass Spec.* **2006**, *20* (6), 1001–1012. DOI: 10.1002/rcm.2401.

(2) Kawano, S.-i.; Inohana, Y.; Hashi, Y.; Lin, J.-M. Analysis of keto-enol tautomers of curcumin by liquid chromatography/mass spectrometry. *Chin. Chem. Lett.* **2013**, *24* (8), 685–687. DOI: https://doi.org/10.1016/j.cclet.2013.05.006.

(3) Chatterjee, P.; Dutta, S. S.; Chakraborty, T. Tautomers and Rotamers of Curcumin: A Combined UV Spectroscopy, High-Performance Liquid Chromatography, Ion Mobility Mass Spectrometry, and Electronic Structure Theory Study. *J. Phys. Chem. A* **2022**, *126* (10), 1591–1604. DOI: 10.1021/acs.jpca.1c08612.

(4) Inoue, K.; Nomura, C.; Ito, S.; Nagatsu, A.; Hino, T.; Oka, H. Purification of Curcumin, Demethoxycurcumin, and Bisdemethoxycurcumin by High-Speed Countercurrent Chromatography. *J. Ag. Food Chem.* **2008**, *56* (20), 9328–9336. DOI: 10.1021/jf801815n.

(5) Verma, M. K.; Najar, I. A.; Tikoo, M. K.; Singh, G.; Gupta, D. K.; Anand, R.; Khajuria, R. K.; Sharma, S. C.; Johri, R. K. Development of a validated UPLC-qTOF-MS Method for the determination of curcuminoids and their pharmacokinetic study in mice. *DARU J. Pharm. Sci.* **2013**, *21* (1), 11. DOI: 10.1186/2008-2231-21-11.
